# The regulatory role of pioneer factors during cardiovascular lineage specification – A mini review

**DOI:** 10.3389/fcvm.2022.972591

**Published:** 2022-08-23

**Authors:** Javier E. Sierra-Pagan, Daniel J. Garry

**Affiliations:** ^1^Cardiovascular Division, Department of Medicine, University of Minnesota, Minneapolis, MN, United States; ^2^Stem Cell Institute, University of Minnesota, Minneapolis, MN, United States; ^3^Paul and Sheila Wellstone Muscular Dystrophy Center, University of Minnesota, Minneapolis, MN, United States

**Keywords:** pioneer factors, ETV2/ER71, ETS factors, cardiac regeneration, chromatin remodeling, reprogramming, epigenetics

## Abstract

Cardiovascular disease (CVD) remains the number one cause of death worldwide. Ischemic heart disease contributes to heart failure and has considerable morbidity and mortality. Therefore, alternative therapeutic strategies are urgently needed. One class of epigenetic regulators known as pioneer factors has emerged as an important tool for the development of regenerative therapies for the treatment of CVD. Pioneer factors bind closed chromatin and remodel it to drive lineage specification. Here, we review pioneer factors within the cardiovascular lineage, particularly during development and reprogramming and highlight the implications this field of research has for the future development of cardiac specific regenerative therapies.

## Introduction

Cardiovascular disease is the number one cause of death in the U.S and worldwide. Vascular and cardiac disease results in considerable morbidity and mortality (1, 2). The only curative disease for end-stage cardiovascular disease is orthotopic heart transplantation (3). While it is estimated that more than 100,000 Americans could benefit from cardiac transplantation, only 3,000 to 3,500 recipients receive such therapy due to limited donor organ availability (4). Therefore, new therapies are warranted.

Reprogramming of lineages has received intense interest and several outstanding reviews are available and provide a comprehensive overview of the field (5, 6). This field had its genesis, in part, based on the discovery of master regulators—those factors that promote lineage specific gene expression when overexpressed in somatic cells such as fibroblasts (6). These assays were referred to as conversion assays and the first master regulator to be described was Myod and its family members (7). Subsequently, more than 200 master regulators have been described and their functional roles have been explored using gene disruption technologies (8).

While the role(s) of master regulators focused primarily on their ability as transcription factors to govern lineage specific gene expression, pioneer factors function to bind nucleosomal DNA and relax the chromatin landscape upstream of lineage specific genes (6, 8, 9). In this fashion, pioneer factors reside at the very top of the hierarchical molecular cascade. Moreover, there are only a limited number of rigorously defined pioneer factors (9). Here, we highlight the criteria necessary for inclusion as a pioneer factor, we provide an overview of the field itself and highlight the role and mechanisms of pioneer factors that govern the cardiovascular field.

## Coordinated role of networks and lineage specification during cardiovascular development

**Figure 1:**
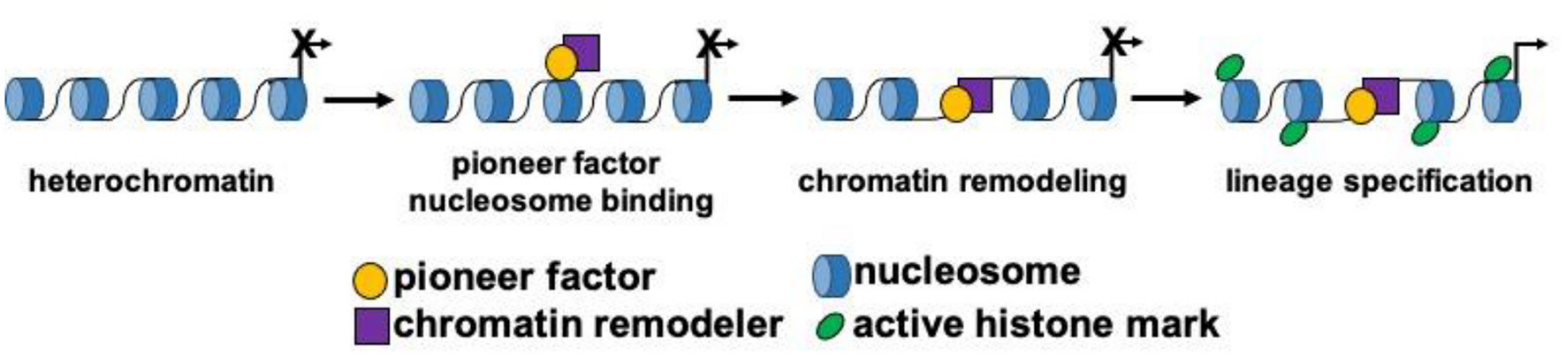
Pioneer factors drive lineage specification. Schematic model depicting the function of pioneer transcription factors during cellular lineage specification. Pioneer factors initially bind to nucleosomal DNA and then remodel chromatin by themselves or by recruiting a chromatin remodeling factor. These steps lead to the activation of gene expression and changes to the epigenetic landscape surrounding the DNA binding sites of the pioneer factor.

Cardiovascular development is a well-coordinated and complex process that requires the specification, proliferation, migration and differentiation of progenitor cells that become coupled to form a functional syncytium within the heart (10–14). Progenitor cells arising from the mesodermal germ layer form the early cardiac crescent and fuse to form the linear heart tube. Progenitor cell populations and their derivatives contribute to the first and second heart fields. These respective progenitor cell populations contribute to distinct structures within the mature heart, and are combinatorily regulated by distinct and overlapping transcriptional networks (15). Stage specific transcription factors and signaling pathways have been defined and function as key regulators of cardiovascular development. As previously outlined, master regulatory genes govern the transcriptional cascades and direct cellular lineages during differentiation and cellular reprogramming. However, within this group of master regulators, a small subset of transcription factors known as pioneer factors, have the unique capacity to bind and remodel silent and compacted regions of chromatin (nucleosomal DNA) to drive the expression of lineage specific genes that allow for development and reprogramming to occur (Figure 1). Due to their unique capacity to bind nucleosomal DNA and drive lineage development, pioneer factors have been shown to be critical factors for regenerative sciences and cancer biology.

## Role of master regulators during development

Essential transcription factors, known as master regulators, regulate cell fate and lineage commitment development. These master transcription factors regulate lineage commitment events, and can convert/reprogram cells (fibroblasts) to specific lineages (8, 16). In addition to the MYOD family, other prototypic master regulators include Pdx1, an important regulator of pancreas development (17). Global knockout of *Pdx1* in the mouse results in the absence of the pancreas and similar to MYOD, ectopic overexpression of PDX1 converts somatic cells to pancreatic acinar cells (18, 19). Other master regulators have been identified for each lineage including (but not limited to): MESP1, MYF5, NEUROD, ASCL1/MASH1, GATA2, GATA4, PAX3, PAX7, FOXO, FOXA, SCL/TAL1, HIF1 and others (7, 17, 20–24). These lineage specific transcription factors or master regulators are essential for the different combinations of reprogramming factors used to develop organ specific cellular therapies (6). Among these master regulators, a subset of transcription factors known as pioneer factors, initiate lineage specific regulatory events to open up or relax compacted (heterochromatic) DNA to govern developmental and reprogramming processes (6, 25, 26) (Figure 1).

## Role of pioneer factors during lineage specification

Pioneer factors are a specialized group of lineage-specific transcription factors that bind heterochromatic regions of DNA to promote chromatin relaxation and recruit non-pioneer transcription factors for lineage development or reprogramming to occur (Figure 1) (9, 25, 26). This important functional role is due to their unique capacity to scan heterochromatin, recognize partial (non-canonical) DNA motifs that are exposed on the surface on nucleosomes and bind to them. The complexes that are formed (chromatin remodeling and non-pioneer factors) following the binding of a pioneer factor are context dependent (i.e., cell type specific) and serve to dictate the diverse mechanisms whereby pioneer factors can regulate lineage specification and development. Pioneer factors were discovered with the dissection and definition of the mechanisms whereby liver specific regulatory complexes were bound to heterochromatin early during development (27, 28). These studies identified FOXA1 as the first prototypical pioneer factor that regulates hepatic lineage specification during early embryonic development (Table 1) (25, 29). A distinct feature regarding FOXA1 is that its DNA binding domain (*forkhead*/winged helix domain) shares a similar structure to that of linker histones, which enables this pioneer transcription factor to displace linker histones from nucleosomes to remodel chromatin and promote liver development (30, 31). The discovery of FOXA1 as a pioneer factor has led to the identification and characterization of other pioneer factors (Table 1). Perhaps, the most recognized examples of pioneer factors are OCT4, SOX2 and KLF4 (OSK), which promote the reprogramming of terminally differentiated fibroblasts to induced pluripotent stem cells (iPSCs) (32, 33). While c-MYC is also necessary for the reprogramming process of fibroblasts to iPSCs, unlike OSK, which can bind partial DNA motifs in nucleosomal DNA located in enhancers, c-MYC binds accessible regions in promoters and not nucleosomal DNA (25, 32, 33).

**Table 1:** Pioneer factors with established properties.

***Pioneer***	***Lineage (cell)***	***Nucleosome binding***	***Chromatin remodeling***	***Transcription factor cooperation***	***Ref*.**
*ASCL1*	Neuron	Yes	Yes	BRN2	(126–129)
*C/EBPα*	Macrophage	Yes	Yes	PU.1	(119)
*EBF1*	B cell	Yes	Yes	PAX5	(117, 118)
*ETV2*	Endothelial cell	Yes	Yes	ELK3	(37)
*FOXA1, FOXA2*	Liver/Pancreas/Hormone dependent	Yes	Yes	GATA4,C/EBPβ,HNF4α /GATA6/Nuclear receptors	(29, 31, 130–137)
*FOXH1*	Mesendoderm	Yes	Yes	-	(138)
*GRHL1, GRHL2, GRHL3*	Epithelial cell	Yes	Yes	-	(139, 140)
*GAF*	Zygote	Yes	Yes	-	(141)
*ISL1*	Cardiac progenitor cell	Yes	Yes	GATA4	(44)
*KLF4*	iPS cells	Yes	Yes	MYC	(32, 33, 142)
*MYOD1*	Myoblast	Yes	Yes	-	(7)
*PAX7*	Melanotrope	Yes	Yes	TPIT	(34, 39, 143)
*PU.1*	Macrophage/ T cell	Yes	Yes	-	(115, 116, 120–122)
*OCT4*	iPS cells	Yes	Yes	MYC	(32, 33, 142)
*OPA*	Zygote	Yes	Yes	-	(144, 145)
*SOX2*	iPS cells	Yes	Yes	MYC	(32, 33, 142)
*TCF1*	T cell	Yes	Yes	-	(146)
*ZELDA*	Zygote	Yes	Yes	-	(147)

iPS, induced pluripotent stem cell.

Ref., reference.

The molecular mechanisms whereby pioneer factors promote lineage specification and development remain ill-defined. One area of interest in the field is the effect of chromatin modifications (histones or DNA) on pioneer factor binding. A mechanism that has been observed is the binding of pioneer factors to nucleosomal DNA sites without any apparent effect. These regions are termed “pioneer factor resistant sites” and are being examined to define the co-regulatory mechanisms that are present in the chromatin environment to control cell fate by promoting or preventing chromatin remodeling and lineage specification by a pioneer factor following binding (34). For example, the pioneer factor PAX7 is able to bind facultative heterochromatin (H3K9me2) but not constitutive heterochromatin (H3M9me3) in order to regulate pituitary progenitor cell development (33–35). This action highlights the need to characterize the role of different histone and DNA modifying enzymes that regulate pioneer factor function by directly interacting with them or by indirectly modifying the chromatin landscape during lineage specification (33).

A majority of the pioneer factor studies have focused on the characterization of the activation of lineage transcriptional programs and the initial functions of pioneer factors. However, the mechanisms driving lineage repression and the later stages of lineage specification are not well understood and warrant further investigation. ASCL1 is one of the few pioneer factors whose repressive role has been explored and found to recruit cooperating factors such as the myelin transcription factor 1- like protein (MYT1L), which represses the myogenic lineage program during the reprogramming of fibroblasts to neuronal cells (36). Studies on these cooperating or settler factors have demonstrated recruitment of both non-pioneer transcription factors as well as chromatin remodeling enzymes (p300 and BRG1) to allow for lineage specification to occur by activating gene expression, modifying chromatin or stabilizing the binding of pioneer factors to their DNA binding sites (9, 37, 38). Gaining a deeper understanding on how pioneer factor function is regulated by these settler factors may enhance reprogramming strategies to more efficiently drive lineage development in vivo to treat cardiovascular disease (6).

## Role of chromatin modifying factors for the function of pioneer factors

While pioneer factors are required for the initial binding to nucleosomal DNA, cooperation with other (non-pioneer factors) is necessary in order to drive lineage development and reprogramming (9, 25, 39). Two important events are required following the binding of a pioneer factor and these include: (1) chromatin relaxation and (2) recruitment/interaction with other transcription factors (Figure 1). These two events enable the effects of pioneer factors and lineage development to occur by amplifying the signal and providing context dependent mechanisms in different regions of the genome (9, 25). Chromatin relaxation is a crucial step during lineage development where pioneer factors have been shown to promote remodeling by themselves (FOXA1) or with the assistance of the SWI/SNF complex (9, 25). The SWI/SNF complex of proteins is one of the most studied chromatin remodeling complexes. This complex increases DNA accessibility to regulate the development or reprogramming of pluripotent, neuronal, cardiac and endothelial cells (37, 40). SMARCA4 (BRG1), the ATPase subunit of the SWI/SNF complex, is an important regulator of early embryonic development as *Brg1* null embryos are lethal prior to implantation (41). Using in vitro differentiation and mouse studies, BRG1 has also been shown to be an important regulator of cardiovascular development and disease (42, 43). BRG1 also has been shown to be an important chromatin remodeler as it interacts with at least four different pioneer factors (OCT4, GATA3, ISL1 and ETV2) in a context dependent fashion to regulate chromatin remodeling and two of these pioneer factors are important regulators of cardiovascular development (Table 1) (37, 38, 44, 45).

## Pioneer factors in the cardiovascular lineage

The cardiovascular lineage is composed of multiple lineages including: the muscle, vascular/endothelial and hematopoietic lineages (46–51). While many master regulators have been described and have important roles in the coordination of the development of the cardiovascular lineage, few pioneer factors have been identified within this lineage (8, 9). In part, this is due to the complexity associated with the different cellular lineages and structures within the cardiovascular system (13, 52). These pioneer factors are ISL1, GATA4 and ETV2, and in this section we will discuss the data supporting their pioneer role and function in the regulation of the cardiovascular lineage.

ISL1 is an important regulator for the development of the second heart field (SHF), and was recently identified as a pioneer factor (Table 1). *Isl1* KO mice lack the right ventricle, outflow tract and portions of the atria because of its role as an important regulator of SHF cardiac progenitor cells (CPCs) (53–56). In recent studies by Gao et al., they described that ISL1, like other pioneer factors, regulated the development of SHF CPCs by binding nucleosomal DNA and relaxing chromatin by forming a complex with BRG1-BAF60C (44). They also identified GATA4 as a cooperating factor in selected sites bound to ISL1, suggesting a potential interaction for ISL1 in the regulation of cardiovascular development. Together, ISL1 and GATA4 were shown to bind regulatory DNA regions of important cardiac genes such as *Hand2, Myocd, Ttn, Ryr2* and others. The exact mechanism whereby GATA4 promotes the pioneer function of ISL1 in these regulatory regions remains to be elucidated. These studies used both in vivo and in vitro assays to demonstrate that ISL1 binds nucleosomal DNA to regulate SHF development.

GATA4 is another important master regulator of cardiovascular development. Loss of *Gata4* has been shown to lead to early cardiac defects and results in bifed (non-fused) heart fields and embryonic lethality (57–62). Additionally, GATA4 has the capacity (along with other master regulators) to reprogram fibroblasts to induced cardiomyocytes (iCMs) *in vitro* and *in vivo* (63–67). While GATA4 is a key regulator of cardiovascular development, its role as a pioneer factor has only been described in hepatic progenitors and reprogramming of fibroblasts to hepatic-like cells (29, 68). A recent study combined scRNAseq, ATACseq, ChIPseq and machine learning to define the molecular mechanisms governing iCM reprogramming using GATA4, MEF2C and TBX5 (GMT) and concluded that MEF2C and TBX5, but not GATA4 bind heterochromatin and promote chromatin remodeling during reprogramming (69). While these studies do not support the notion that GATA4 is a pioneer factor for the cardiac muscle lineage, further developmental studies are necessary to understand the heterochromatin binding and chromatin remodeling capabilities of GATA4 during cardiovascular development. The recent ISL1 studies suggest that GATA4 may bind to heterochromatin in the cardiovascular lineage independently but more mechanistic studies are needed (44).

More recently, ETV2 was identified as a pioneer factor for the cardiovascular lineage that regulates and reprograms endothelium (Table 1) (37). ETV2 is an essential transcription factor for the development of endothelial and hematopoietic lineages (51, 70–108). Its expression is observed in mesodermal progenitors and hemangioblasts that give rise to endocardial/endothelial and hematopoietic lineages, while repressing other lineages such as the cardiac and skeletal muscle lineages (49, 74, 79). Key regulatory genes and pathways for the cardiovascular lineage such as MESP1, NKX2-5, Wnt/Notch/BMP signaling (among others) have been shown to regulate ETV2 expression within the cardiovascular lineage (50, 74, 109, 110). Loss of *Etv2* results in lethality by E8.5 in developing mouse embryos due to the lack of all vascular and blood lineages. Moreover, congenital heart defects in aborted developing human fetuses have been reported to harbor *Etv2* mutations (50, 51, 111). Additionally, ETV2 overexpression (alone) reprograms terminally differentiated cells (fibroblasts) to endothelial cells both *in vitro* and *in vivo* (112). Our recent findings characterized the molecular mechanism whereby ETV2 regulates the endothelial lineage as a pioneer factor (37). ETV2 can scan the genome, bind nucleosomal DNA and remodel chromatin independent of its cellular context, whether it is related to fibroblast reprogramming or mouse embryonic stem cell (mESC) differentiation into endothelial progenitor cells (Figure 1). We characterized this functional role for ETV2 using scRNAseq, ATACseq, NOMEseq, ChIPseq and *in vitro* nucleosomal binding assays to unequivocally demonstrate that ETV2 binds nucleosomal DNA during endothelial lineage reprogramming/development. We identified canonical downstream targets for ETV2 such as *Emcn, Lmo2, Rhoj* and others that were bound by ETV2 during endothelial lineage development and reprogramming. Similar to ISL1, ETV2 recruits and directly interacts with BRG1. BRG1 is an essential co-factor for ETV2 to function as a pioneer factor as *Brg1* knockdown and conditional knockout significantly affected the ability of ETV2 to remodel chromatin and drive endothelial lineage formation in both reprogrammed fibroblasts and differentiating mESCs, respectively (37). This ETV2-BRG1 interaction was verified using mass spectrometry, Co-IP assays and GST-pulldown assays. Additionally, we demonstrated that this interaction was important for enacting epigenetic changes during endothelial lineage development such as the deposition of histone 3 lysine 27 acetylation (H3K27Ac) in regions surrounding ETV2-BRG1 bound sites. Lastly, by screening co-factors we identified ELK3 as a transcription factor that was recruited to ETV2-BRG1 bound sites following chromatin remodeling and ELK3 has an important role in endothelial cell development (Figure 1 and Table 1). Understanding how other factors might regulate chromatin remodeling and the pioneer activity of ETV2, such as FOXC2 which is known to regulate Fox-Ets enhancer motifs during endothelial lineage development in combination of ETV2, will be important for further dissecting this molecular mechanism (113). Forkhead transcription factors are important in the field of pioneer factors and chromatin remodeling because of their unique protein structure that resembles linker histones and allows (some of the *forkhead* family members) to remodel chromatin (31). Furthermore, the expression of ETV2 is transient during development, understanding how other downstream co-factors (i.e., ELK3, FLI1, SCL/TAL1, etc.) direct the developmental machinery following the downregulation of ETV2 will be important for the development of therapeutic strategies using ETV2 to develop mature vasculature that can be used for ischemic diseases such as the transplantation of human vasculature (71, 114).

ETV2 possesses an Ets DNA binding domain (DBD) characterized by a winged helix-turn-helix structure which needs to be studied in terms of how it interacts with nucleosomal DNA to allow for chromatin binding and remodeling to occur (75). Previous studies on *forkhead* factors have demonstrated that the winged helix DBD of the pioneer factor FOXA resembles that of the structure of linker histones, while Ets factors have been shown to use their short α-helix structure to bind the major groove of DNA to target nucleosomes (25). We hypothesize that the winged helix of ETV2 will most likely behave like that of previously described Ets factors, but it remains to be explored.

Unlike ISL1 and GATA4, the pioneer function of ETV2 in the cardiovascular lineage is independent of its cellular context, whether it is cellular differentiation or reprogramming, it functions in a similar fashion in both model systems. Future studies will need to focus on further characterization of the molecular mechanisms driving endothelial cell development/reprogramming by ETV2 to enhance therapeutic approaches to develop mature vasculature for ischemic diseases. While ETV2 is an essential regulator of hematopoietic development, we did not define ETV2 as a pioneer factor for hematopoietic lineages and therefore we hypothesize that other co-factors and pioneer factors might facilitate this developmental process. For example, EBF1, PU.1 and C/EBPα regulate hematopoietic development and act as pioneer factors for the B cells, DN3 t cells and macrophages (115–122) (Table 1). Whether these factors are regulated by ETV2 early on or they act independently of ETV2 to regulate the development of hematopoietic lineages remains to be elucidated. Identifying this pioneer role for ETV2 has big implications for the development of regenerative therapies that aim to generate vasculature for ischemic diseases, particularly in the cardiovascular system (71, 112). Additionally, although not the focus of this review, the development of therapies that target pioneer factors in cancer will be very important. As ETV2 has been shown to have a role in cancer (82, 88, 98, 123), understanding whether its ability to remodel silent/compacted chromatin as a pioneer factor has important implications during angiogenesis, as therapeutic initiatives could target and inhibit ETV2 thereby impacting tumorigenesis (82, 98, 123). Additionally, it would be interesting to determine whether or not BRG1 or another chromatin remodeler also forms a complex with ETV2 in the context of cancer.

## Conclusion(s)

More studies are emerging that claim to have characterized a novel pioneer transcription factor and this number will be expected to increase (8). This is in part due to the advances in molecular biology that facilitate the cellular characterization at the single cell level during embryogenesis. Further, these technologies will allow us to identify DNA binding sites for transcription factors (TF) and more importantly allow us to define the chromatin dynamics surrounding the DNA binding sites of such TFs. Importantly, the development of the Assay for Transposase-Accessible Chromatin followed by sequencing (ATAC-seq) allows for the definition of the chromatin landscape of differentiating or reprogramming cells using very few cells (50,000 cells or less) and support the claim that a TF is a pioneer factor (124, 125). While ATAC-seq characterization of cell populations can be insightful, we caution the reader that a more in-depth analysis is needed when assigning the role of pioneer factor. To designate a pioneer factor, three criteria need to be fulfilled: (1) pioneer factors need to bind nucleosomal DNA *in vivo* (sequencing) and *in vitro* (nucleosomal binding assay), (2) pioneer factors need to promote chromatin remodeling around DNA binding sites by themselves or by interacting with chromatin remodelers and (3) pioneer factors need to enact global epigenetic changes (i.e., demethylation) and recruit other co-factors that further promote the development or reprogramming of a cellular lineage (Figure 1; Table 1).

Further studies will be needed within the cardiovascular field to identify pioneer factors that regulate distinct cellular lineages and structures (i.e., first vs. second heart field) that comprise the four chambered organ. For example, while ETV2 sits at the top of the endothelial lineage developmental hierarchy, ISL1 and GATA4 are two of many regulators of the cardiac muscle lineage with very specific functions. We predict that multiple pioneer factors will be required to regulate cardiac muscle development and reprogramming. Other cellular lineages that were not discussed include: smooth muscle and cardiac fibroblasts as no pioneer factors have been identified for these lineages. Pioneer factors can be powerful tools for the development of regenerative therapies whose goal is to generate mature and functional cell lineages. Understanding the molecular mechanisms that drive lineage development by these and other pioneer factors within the cardiovascular lineage will be instrumental because coupling these pioneer factors along with chromatin remodelers and downstream targets genes can amplify the molecular effect needed to better develop regenerative therapies for cardiovascular disease.

## Author contributions

JS-P and DG: conceived the review and wrote the manuscript. All authors contributed to the article and approved the submitted version.

## Funding

We are grateful for the support of the Department of Defense (Grant # 3002-11765-00089113).

## Conflict of interest

The authors declare that the research was conducted in the absence of any commercial or financial relationships that could be construed as a potential conflict of interest.

## Publisher's note

All claims expressed in this article are solely those of the authors and do not necessarily represent those of their affiliated organizations, or those of the publisher, the editors and the reviewers. Any product that may be evaluated in this article, or claim that may be made by its manufacturer, is not guaranteed or endorsed by the publisher.
